# FASN negatively regulates p65 expression by reducing its stability via Thr^254^ phosphorylation and isomerization by Pin1

**DOI:** 10.1016/j.jlr.2024.100529

**Published:** 2024-03-10

**Authors:** Lincoln Barlow, Sophia Josephraj, Boqing Gu, Zizheng Dong, Jian-Ting Zhang

**Affiliations:** 1 Department of Pharmacology and Toxicology, Indiana University School of Medicine, Indianapolis, IN, USA; 2 Department of Cell and Cancer Biology, University of Toledo College of Medicine and Life Sciences, Toledo, OH, USA

**Keywords:** FASN, isomerization, NF-κB, p65, phosphorylation, Pin1, proteasome

## Abstract

FASN, the sole cytosolic enzyme responsible for de novo palmitate synthesis in mammalian cells, has been associated with poor prognosis in cancer and shown to cause drug and radiation resistance by upregulating DNA damage repair via suppression of p65 expression. Targeting FASN by repurposing proton pump inhibitors has generated impressive outcomes in triple-negative breast cancer patients. While p65 regulation of DNA damage repair was thought to be due to its suppression of poly(ADP-ribose) polymerase 1 gene transcription, the mechanism of FASN regulation of p65 expression was unknown. In this study, we show that FASN regulates p65 stability by controlling its phosphorylation at Thr^254^, which recruits the peptidyl-prolyl *cis/trans* isomerase Pin1 that is known to stabilize many proteins in the nucleus. This regulation is mediated by palmitate, the FASN catalytic product, not by FASN protein per se. This finding of FASN regulation of p65 stability via phosphorylation of Thr^254^ and isomerization by Pin1 implicates that FASN and its catalytic product palmitate may play an important role in regulating protein stability in general and p65 more specifically.

FASN is the primary enzyme responsible for de novo palmitate synthesis in the cytosol. Expression and activity of FASN is generally low in normal nonlipogenic tissues as the modern diet provides a sufficient supply of fatty acids. However, FASN is significantly upregulated in many forms of human cancer as the increased lipid synthesis from FASN activity is essential for the membrane production required for highly proliferating tumor cells (1). Furthermore, high levels of FASN expression may confer a survival advantage for cancer cells and associate with poor prognosis and disease recurrence (2, 3). Indeed, FASN upregulation causes drug resistance (4, 5), which may contribute to poor prognosis.

The nuclear factor kappa B (NF-κB), consisting of RelA/p65 and p50, represents a well-known molecule in a complex signaling pathway that plays important roles in cancer and inflammation (6). The relationship between NF-κB and cancer is multifaceted. The most well-documented role of NF-κB signaling in cancer involves constitutive NF-κB activation and, specifically, the ability of NF-κB to regulate a myriad of genes that control cell proliferation, as well as apoptosis and cell survival (6, 7, 8, 9). Indeed, inhibiting NF-κB activity reversed the tumorigenic phenotype resulting from constitutive NF-κB activation and apoptosis (10, 11). However, it has also been shown that NF-κB activation can promote cell death. Specifically, NF-κB activation mediates tumor TRAIL-induced apoptosis in epithelial carcinoma κB cells and keratinocytes (12), doxorubicin-induced apoptosis in neuroblastoma cells (13), UVB radiation-induced apoptosis (14), and paclitaxel-induced cell death (15).

Recently, it was shown that FASN-induced resistance to doxorubicin and DNA damage is due to suppression of NF-κB expression, resulting in upregulation of poly(ADP-ribose) polymerase 1 (PARP1) expression and nonhomologous end-joining repair of DNA damage (16) and inhibition of TNFα activation of neutral sphingomyelinase for production of ceramide (5). Intriguingly, it was also shown that p65 inhibits PARP1 expression by binding to its promoter sequence. However, how FASN inhibits p65 expression remains unknown. In this study, we determined the mechanism of FASN regulation of p65 expression and found that FASN, via its catalytic product palmitate, negatively regulates the stability of p65 protein involving the phosphorylation of its Thr^254^ and isomerization by the peptidyl-prolyl *cis/trans* isomerase Pin1.

## Materials and methods

### Materials

Pin1 siRNA and Protein G PLUS-Agarose beads were purchased from Santa Cruz Biotechnology, Inc (Dallas, TX). Opti-MEM reduced serum media, normal horse serum, Lipofectamine RNAiMAX reagent, and Lipofectamine 3000 were purchased from Thermo Fisher Scientific, Inc (Waltham, MA). Scrambled control siRNA and antibodies against p65 (D14E12; 1:3,000 dilution for Western blot and 1:50 dilution for immunofluorescence), pSer^536^p65 (93H1; 1:1,000 dilution), ubiquitin (3,936; 1:1,000 dilution), PARP1 (9,542; 1:1,000 dilution), and Pin1 (3,722; 1:1,000 dilution) were from Cell Signaling Technology, Inc. (Danvers, MA). Antibodies against M2-FLAG tag (F1804; 1:1,000 dilution) and β-Actin (A5316; 1:5,000 dilution), secondary antibodies conjugated with HRP, and cycloheximide were all purchased from Sigma-Aldrich, Inc. (St Louis, MO), whereas High-Capacity Complementray DNA (cDNA) Reverse Transcription Kit and SYBR Green PCR Mix were from Applied Biosystems (Waltham, MA). PureLink RNA Mini Kit, Alexa Fluor plus 488 secondary antibody, and donkey anti-Rabbit IgG (A32790, 1:200 dilution) were both from Invitrogen (Waltham, MA). FASN antibody (610,962; 1:1,000 dilution), Dual-Luciferase Assay System, and pCMV3-FLAG-Pin1 plasmid (HG10282-NF) were purchased from BD Biosciences (Franklin Lakes, NJ), Promega Corporation (Madison, WI), and Sino Biological (Wayne, PA), respectively. All other chemicals were purchased from either Sigma-Aldrich or Thermo Fisher Scientific, Inc.

### Cell lines and transient transfections

Human breast cancer cell lines MDA-MB-468 and MDA-MB-231 were cultured at 37°C with 5% (v/v) CO_2_ in DMEM supplemented with 10% (v/v) FBS. The drug-resistant MCF7/AdVp3000 (M3K) cells derived from MCF7 were generated previously (17) and cultured in DMEM supplemented with 10% (v/v) FBS and 3,000 ng/ml adriamycin and 5 μg/ml verapamil to maintain their drug resistance phenotype. Stable FASN-overexpressing MCF7 cells (MCF7/FASN) and corresponding vector-transfected control cells (MCF7/Vec), as well as M3K cells with stable FASN knockdown (M3K/sh(FASN)) and corresponding scrambled shRNA-transfected control cells (M3K/Scr), were generated previously (4) and maintained in DMEM supplemented with 10% (v/v) FBS and 400 μg/ml G418. MDA-MB-436 cells with FASN overexpression (MDA-MB-436/FASN) and its control vector-transfected cells were also generated previously (18) and maintained in MEM supplemented with 10% (v/v) FBS and 300 μg/ml G418.

For transient knockdown using siRNA, cells were plated in 6-well plates and cultured for 24 h, followed by transfection with scrambled control siRNA (Cell Signaling) or siRNA against FASN or Pin1 in Opti-MEM reduced serum media using Lipofectamine RNAiMAX reagent according to the manufacturer’s instructions. For transient overexpression, 2 × 10^5^ cells/well were seeded in 6-well plates for 24 h followed by transfection with pcDNA3.1-FASN and the control pcDNA3.1 vector or pCMV3-FLAG-Pin1 and the control pCMV3 vector using Lipofectamine 3000 as previously described (4, 16). At 48 h following transfection, cells were harvested for Western blot analyses.

### Establishment of stable FASN knockdown of MDA-MB-468 cells

For generating FASN knockdown stable clones, cells were transfected with scrambled control and shRNA targeting FASN were transfected with Lipofectamine 3000 per the manufacturer’s protocol. After 24 h of transfection, cells were trypsinized and seeded in 100 mm dish and cultured overnight. The cells were then treated with 800 μg/ml G418 until development of individual colonies. Clones were selected and expanded for further analysis.

### Western blot analysis

Western blot analysis was performed as previously described (16) using primary antibodies against FASN, p65, pSer^536^p65, pIkKb**α**, PARP1, M2-FLAG, ubiquitin, Pin1, and β-Actin, and secondary antibodies conjugated with HRP. Signals were developed using enhanced chemiluminescence, captured using X-ray film, and quantified using ImageJ (National Institutes of Health, Bethesda, MD).

### Real-time RT-PCR analysis

Quantitative real-time RT-PCR analysis was performed also as previously described (5, 16, 19). Briefly, total RNAs were isolated using PureLink RNA Mini Kit and reverse-transcribed to cDNA using High Capacity cDNA Reverse Transcription Kit followed by real-time RT-PCR analysis using SYBR Green PCR Mix and primers 5′-TCTCCCTGGTCACCAAGGAC-3′ (forward) and 5′-TCATAGAAGCCATCCCGGC-3′ (reverse) for p65, 5′-CCCAGGCAGTCAGATCATCTTC-3′ (forward) and 5′-GGTTTGCTACAACATGGGCTACA-3′ (reverse) for TNF-α, 5′-GCTGACCCCAGGCTGTGA-3′ (forward) and 5′-TGCTCCATGTCCGTGAACTG-3′ (reverse) for FASN, 5′-TGATAGCAGCAAGGATCCCAT-3′ (forward) and 5′-CCGTGCCACAGCAATCTTCG-3′ (reverse) for PARP1, and 5′-AAGGACTCATGACCACAGTCCAT-3′ (forward) and 5′-CCATCACGCCACAGTTTCC-3′ (reverse) for GAPDH. GAPDH house-keeping gene was used as an internal control. Relative target gene expression level was calculated using threshold cycles (C_T_).

### NF-κB reporter activity assay

NF-κB cis-reporter assay was performed according to the manufacturer’s instructions using the Path Detect cis-reporting system (Agilent) as described previously (16). Briefly, cells were seeded and cultured overnight before cotransfection with pNF-κB cis-reporter plasmid expressing firefly and pRL-TK renilla luciferase control plasmid for transfection efficiency. An empty vector excluding the NF-κB elements was also transfected as a negative control for reporter activity. At 48 h following transfection, cells were harvested and luciferase activity was determined using the Dual-Luciferase Assay System. NF-κB activity was calculated by normalizing firefly luciferase activity to renilla luciferase activity.

### Immunofluorescence staining of p65

Cells cultured on coverslips with ∼80% confluency were fixed in 4% paraformaldehyde, washed with PBS, permeabilized, and blocked using 10% normal horse serum and 0.2% Triton X-100 in PBS, followed by incubation with anti-p65 antibody in PBS containing 1% horse serum and 0.3% Triton X-100 at 4°C overnight. The cells were then washed with PBS and incubation with Alexa Fluor Plus 488 secondary antibody in PBS containing 1% BSA and 0.3% Triton X-100 for 2 h, followed by 4',6-diamidino-2-phenylindole counterstaining and viewing/imaging using an Olympus 2 FV-1000 MPE inverted confocal microscope.

### [^35^S]-methionine pulse-chase assay

P65 half-life was determined using [^35^S]-methionine pulse-chase labeling as previously described (20, 21). Briefly, M3K/sh(FASN) and control M3K/Scr cells were cultured to 75% confluence before labeling with 10 μCi/ml [^35^S]-methionine for 2 h in methionine-free DMEM. The radioactive media were then removed, and cells were chased in complete DMEM for various times up to 32 h. At different times with 8 h intervals, cells were harvested and subjected to immunoprecipitation using p65 antibody and Protein G PLUS-Agarose beads. The precipitates were separated using SDS-PAGE, and signals were captured using X-ray film. The [^35^S]-methionine-labeled p65 band was excised from the gel and quantified using scintillation counting. Curves for protein degradation over time were generated using one-phase exponential decay in GraphPad Prism (GraphPad Software, Inc) for half-life determination.

### Cycloheximide chase assay

M3K/Sh(FASN) cells or M3K/Scr cells were plated in 6-well plates and cultured for 24 h to reach 70% confluence. Cells were then transfected with either pcDNA(3.1) empty vector, pcDNA(3.1)-FLAG-p65, or pcDNA(3.1)-FLAG-T^245^Ap65 in Opti-MEM reduced serum media using Lipofectamine 3000 (Thermo Fisher) according to the manufacturer’s instructions. At 24 h following transfection, cells were treated with 60 μg/ml cycloheximide for various times up to 12 h before harvest for Western blot analysis of FLAG-p65.

### Ubiquitination assay

Cells were plated in 6-well plates and cultured for 24 h to reach 70% confluence before transfection with pcDNA(3.1)-FLAG-p65 plasmid in Opti-MEM reduced serum media using Lipofectamine 3000 according to the manufacturer’s instruction. At 24 h after transfection, cells were treated with 2 μM MG132 for 4 h, followed by lysis of cells and immunoprecipitation of FLAG-p65 using M2-FLAG antibody and Protein G-PLUS Agarose beads and Western blot analysis probed using ubiquitin antibody.

### Statistical analysis

Student’s *t-*test or one-way ANOVA with Tukey post hoc test was used for statistical analysis where applicable, and *P* <0.05 was considered statistically significant. Results are presented as mean ± standard deviation. For statistical calculations, at least three independent experiments were performed. Statistical analysis calculations were performed using Excel and GraphPad Prism.

## Results

### FASN inhibits p65 expression

**Fig. 1:**
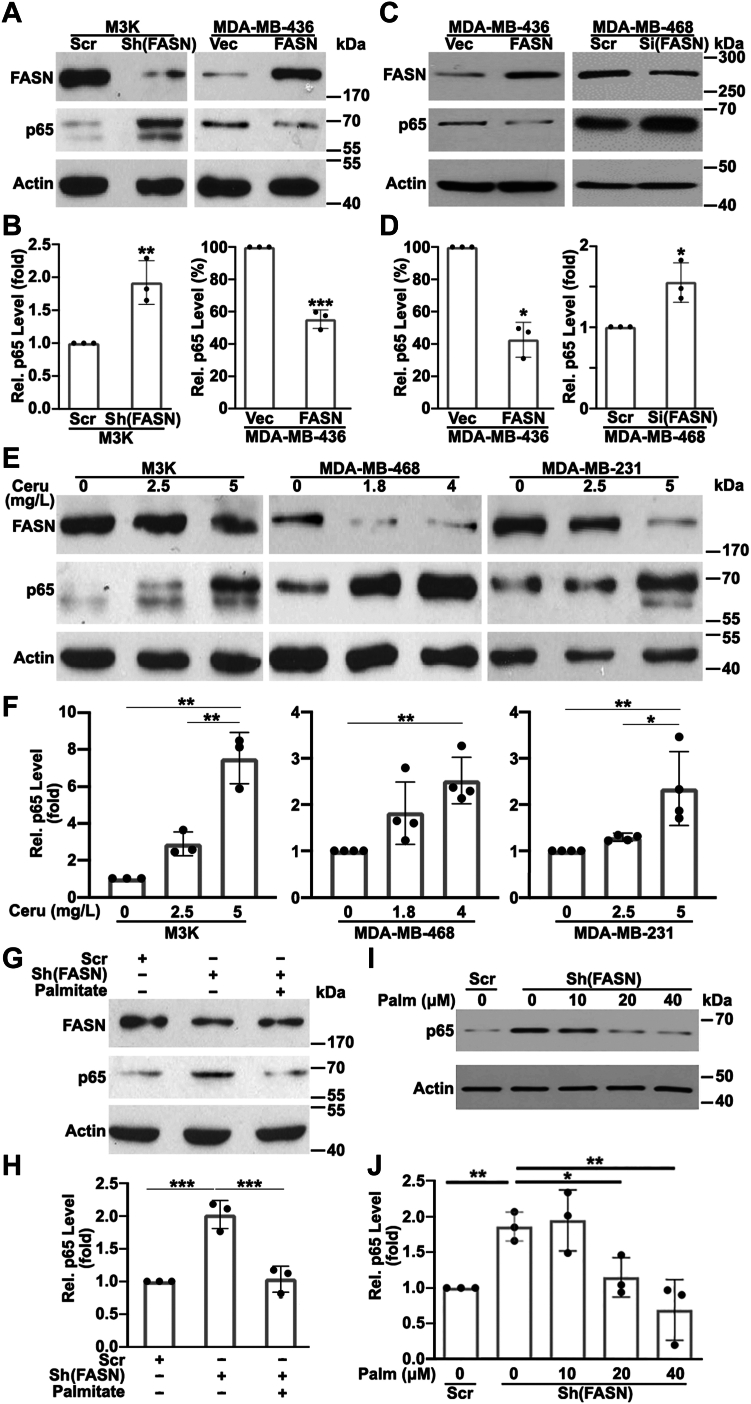
FASN regulation of p65 expression. A, B: Western blot analysis of FASN, p65, and actin loading control in stable M3K/Sh(FASN) and MDA-MB-436/FASN cells compared with their respective control M3K/Scr and MDA-MB-436/Vec cells. C, D: Western blot analysis of FASN, p65, and actin loading control in MDA-MB-436 cells transiently transfected with vector control (Vec) or FASN cDNA or in MDA-MB-468 cells transiently transfected with scrambled control (Scr) or FASN siRNAs. E, F: Western blot analysis of FASN, p65, and actin loading control in M3K, MDA-MB-468, and MDA-MB-231 cells treated with increasing concentrations of cerulenin (Ceru) for 72 h. G, H: Western blot analysis of FASN, p65, and actin loading control in M3K/Sh(FASN) and control M3K/Scr cells following treatment without or with 30 μM exogenous palmitate (Palm) for 48 h. I, J: Western blot analysis of p65 and actin control in MDA-MB-468 cells with stable FASN knockdown following treatments with different concentrations of palmitate. B, D, F, H, J: Quantifications of p65 proteins from Western blots in A, C, E, G, and I, respectively, of ≥3 independent experiments each (∗*P* < 0.05; ∗∗*P* < 0.01; ∗∗∗*P* < 0.001).

To determine the mechanism of FASN regulation in p65 expression, we first tested p65 expression using Western blot analysis of previously established stable clones with FASN knockdown in drug-resistant M3K cells (M3K/Sh(FASN)) derived from MCF7 and with FASN overexpression in MDA-MB-436 cells (MDA-MB-436/FASN). As shown in Fig. 1A, B, p65 expression is indeed significantly increased in the FASN knockdown and decreased in the FASN overexpression cells compared with their respective control cells (M3K/Scr and MDA-MB-436/Vec). To validate these findings, we transiently overexpressed FASN in MDA-MB-436 cells, which also led to p65 downregulation (Fig. 1C, D). Furthermore, transient FASN knockdown in another breast cancer cell line, MDA-MB-468, consistently upregulated p65 expression (Fig. 1C, D). Thus, FASN regulation of p65 expression is independent of acute or chronic change of FASN expression or cell lines used.

Next, we investigated if FASN activity is responsible for regulating p65 expression using Western blot analysis of p65 expression following treatment of M3K, MDA-MB-468, and MDA-MB-231 cells with cerulenin, an inhibitor of FASN activity (22, 23), for 72 h at different concentrations referenced from its IC_50_ in these cells (supplemental Fig. S1). As shown in Fig. 1E, F, cerulenin treatment increased p65 expression in a concentration-dependent manner in all three cell lines. Because cerulenin treatment for 72 h also reduced FASN expression likely because of cerulenin binding-induced destabilization of FASN, we tested shorter treatment to eliminate the possible effect of FASN reduction on p65. As shown in supplemental Fig. S2, cerulenin treatment for 2 h significantly increased p65 expression but without influencing FASN expression. Thus, cerulenin effect on p65 is unlikely because of FASN expression downregulation but because of inhibition of its activity.

Consistently, treatment with lansoprazole, which has been shown to effectively inhibit FASN activity (24, 25), also increased p65 expression in these cells without any effect on FASN expression (supplemental Fig. S3). Based on above observations, we believe that the catalytic activity of FASN is likely responsible for decreasing p65 expression.

Finally, we tested the possibility that palmitate, the catalytic product of FASN, mediates FASN suppression of p65 expression by supplementing palmitate in media for M3K/Sh(FASN) cells before examining p65 expression using Western blot. As shown in Fig. 1G, H, the increase in p65 expression because of FASN knockdown was reversed by palmitate supplementation. Furthermore, this reversal is dependent on the concentration of palmitate used (Fig. 1I, J). Hence, the production of palmitate catalyzed by FASN likely mediates FASN suppression of p65 expression.

### FASN inhibits NF-κB activity

**Fig. 2:**
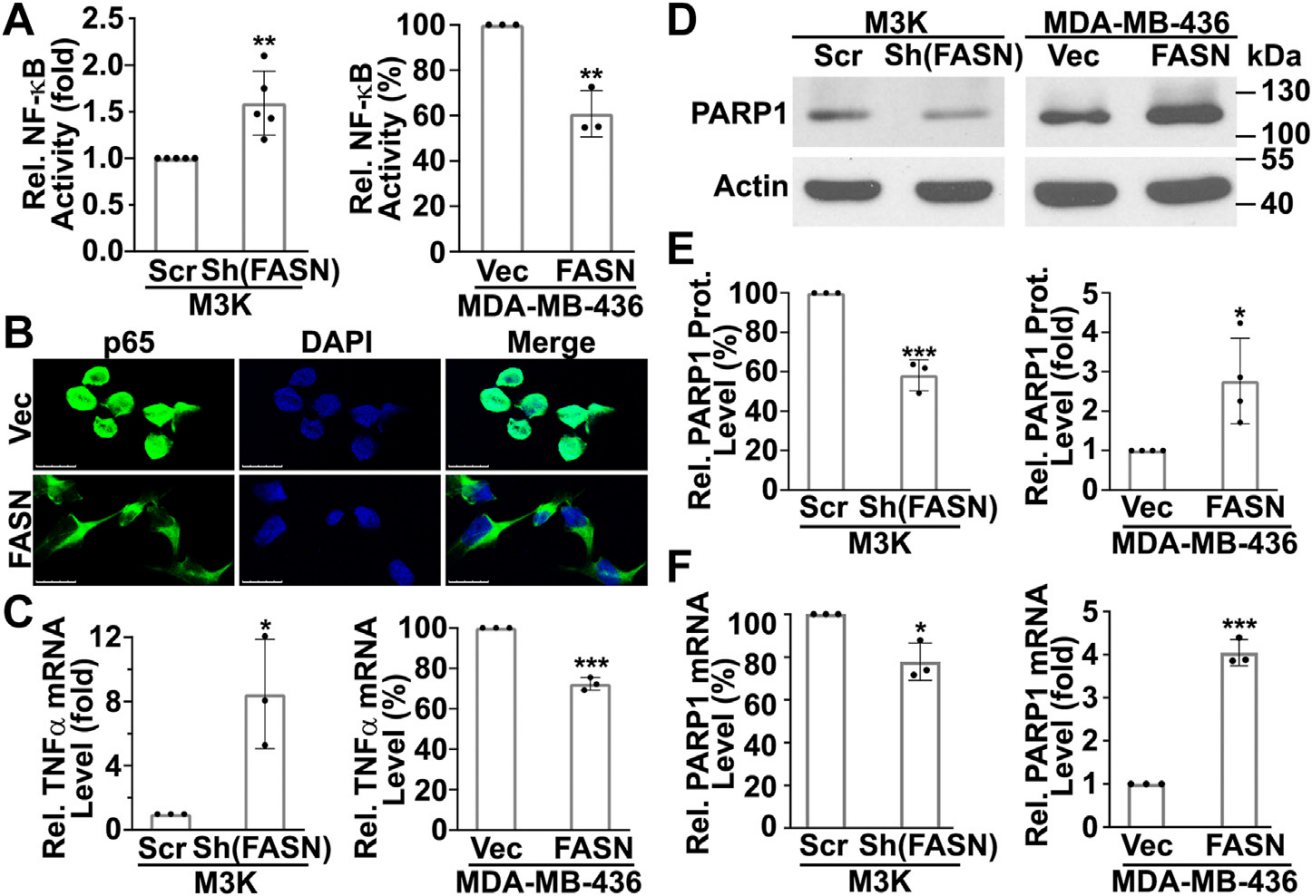
FASN regulation of NF-κB activity. A: Effect of FASN expression on reporter activity driven by NF-κB. Stable M3K/Sh(FASN) and MDA-MB-436/FASN cells with their respective control M3K/Scr and MDA-MB-436/Vec cells were transfected with NF-κB cis-reporter plasmid expressing the firefly luciferase gene along with a pRL-TK renilla luciferase plasmid as a control for transfection efficiency. At 48 h after transfection, dual luciferase activity assay was performed to measure luciferase gene expression as an output of NF-κB activity. B: Immunofluorescence staining of p65 in MDA-MB-436/FASN and its control MDA-MB-436/Vec cells. Scale bar represents 25 μm. C–F: Effect of FASN on TNF-α and PARP1 expression. Stable M3K/Sh(FASN) and MDA-MB-436/FASN cells along with their respective control M3K/Scr and MDA-MB-436/Vec cells were subjected to analyses of TNF-α and PARP1 mRNA using real-time RT-PCR (C, F) or PARP1 protein using Western blot (D, E). E: Quantification of Western blot data shown in D from ≥3 independent experiments (∗*P* < 0.05; ∗∗*P* < 0.01; ∗∗∗*P* < 0.001).

To determine if FASN regulation of p65 expression results in alteration in NF-κB activity, we first performed a reporter assay of NF-κB activity in the stable cells with altered FASN expression. As shown in Fig. 2A, NF-κB activity was significantly increased in M3K/Sh(FASN) cells and significantly reduced in MDA-MB-436/FASN cells compared with their respective control M3K/Scr and MDA-MB-436/Vec cells. Furthermore, less p65 is localized in the nucleus in MDA-MB-436/FASN cells compared with the MDA-MB-436/Vec cells (Fig. 2B), consistent with reduced p65 expression and NF-κB activity in MDA-MB-436/FASN cells.

To validate the above findings and eliminate potential issues with the use of exogenous reporter construct, we determined the expression of endogenous NF-κB downstream target genes, TNF-α and PARP1, using quantitative RT-PCR and Western blot analyses, respectively. As shown in Fig. 2C, FASN knockdown in M3K cells increased, whereas FASN overexpression in MDA-MB-436 cells suppressed the expression of TNF-α. Consistently, FASN knockdown reduced, whereas FASN overexpression increased PARP1 expression at both the protein and mRNA levels (Fig. 2D–F). Taken together, we conclude that FASN regulates p65 expression and NF-κB activity in breast cancer cells.

### FASN does not regulate p65 gene transcription

**Fig. 3:**
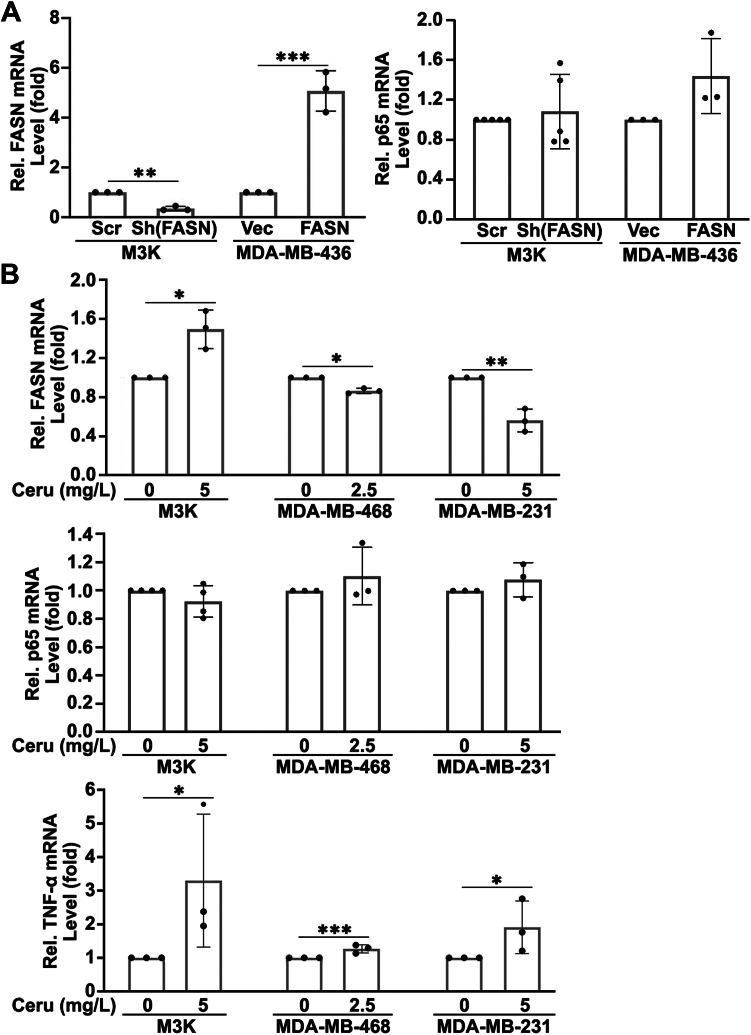
Effect of FASN expression or inhibition on p65 mRNA level. A: Real-time RT-PCR analyses of FASN and p65 mRNA level in stable M3K/Sh(FASN) and MDA-MB-436/FASN and their respective control M3K/Scr and MDA-MB-436/Vec cells. B: Real-time RT-PCR analyses of FASN, p65, and TNF-α mRNA level in M3K, MDA-MB-468, and MDA-MB-231 cells treated for 72 h without or with 5 mg/l, 2.5 mg/l, or 5 mg/l cerulenin, respectively. B: All quantifications were from ≥3 independent experiments (∗*P* < 0.05; ∗∗*P* < 0.01; ∗∗∗*P* < 0.001).

To determine at which level FASN regulates p65 expression, we first performed quantitative RT-PCR analyses of p65 mRNA in M3K/Sh(FASN) and MDA-MB-436/FASN cells compared with their respective control cells. As shown in Fig. 3A, FASN knockdown in M3K cells or FASN overexpression in MDA-MB-436 cells did not change the mRNA level of p65. Furthermore, cerulenin treatment of M3K, MDA-MB-468, and MDA-MB-231 cells also had no significant effect on the mRNA level of p65 (Fig. 3B), whereas the same treatment upregulated p65 protein (Fig. 1E, F). Consistent with the upregulated p65 protein, the mRNA level of TNF-α was also significantly upregulated by cerulenin treatment in all three cell lines as expected (Fig. 3B). Thus, we conclude that FASN likely regulates the p65 expression at the protein level, but not at the mRNA (transcription and/or RNA stability) level, to control its downstream target gene expression.

### FASN destabilizes p65 protein and induces its degradation in proteosomes

**Fig. 4:**
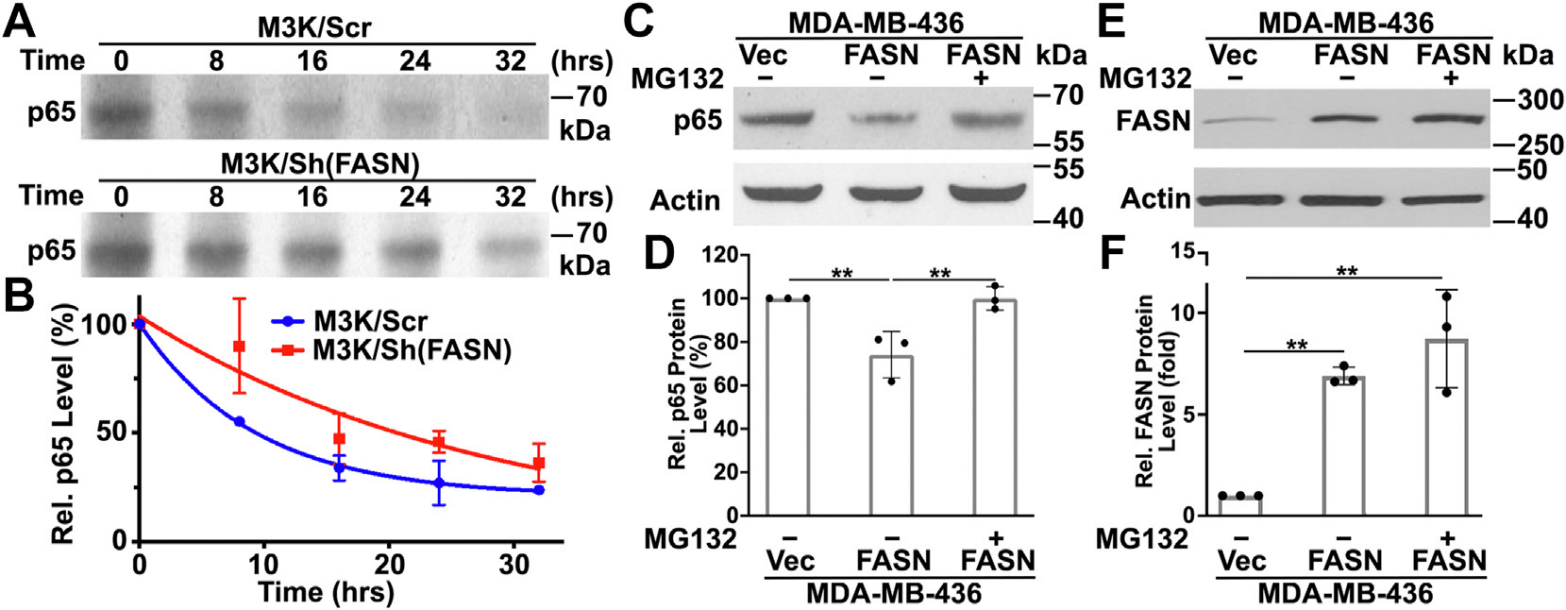
FASN regulation of p65 protein stability. A, B: Effect of FASN knockdown on p65 half-life. Stable M3K/Sh(FASN) and control M3K/Scr cells were pulse-labeled with ^35^S-methionine for 2 h and chased without ^35^S-methionine for various times up to 32 h prior to immunoprecipitation, SDS-PAGE separation, and autoradiography. Data were graphed using one-phase exponential decay for curve fitting using GraphPad Prism. C–F: Effect of MG132 on FASN-induced p65 reduction. Stable MDA-MB-436/FASN and control MDA-MB-436/Vec cells were treated without or with 2 μM MG132 for 24 h followed by Western blot analyses and quantification of p65, FASN, and actin loading control. Quantifications in B, D, and F were from three independent experiments each (∗*P* < 0.05; ∗∗*P* < 0.01).

To determine the possibility that FASN regulates p65 expression at the protein level, we performed a pulse-chase assay to determine the effect of FASN knockdown on the half-life of p65 using M3K/Sh(FASN) and control M3K/Scr cells. As shown in Fig. 4A, B, the half-life of p65 in control M3K/Scr cells is approximately 6 h, which is consistent with that determined in previous studies (26). Interestingly, the half-life of p65 was increased to approximately 19 h in M3K/Sh(FASN) cells. Thus, it is likely that FASN knockdown increased p65 stability.

Previously, it has been shown that p65 degradation occurs in the proteasome (27). To determine the role of the proteasome in FASN regulation of p65 stability, we treated MDA-MB-436/FASN cells with a proteasome inhibitor, MG132, followed by Western blot analysis of p65 protein. As shown in Fig. 4C, D, MG132 treatment rescued p65 expression from FASN suppression in MDA-MB-436/FASN cells. As a control, we also measured the effect of MG132 on FASN expression. As shown in Fig. 4E, F, MG132 had no significant effect on FASN protein level, suggesting that FASN-induced p65 degradation is likely inhibited by MG132. To validate this finding, we performed the same experiment using a previously established MCF7 cell line with stable FASN overexpression (MCF7/FASN) (16) (supplemental Fig. S4A). As shown in supplemental Fig. S4B, MG132 treatment similarly rescued p65 expression from FASN suppression in MCF7/FASN cells. Thus, FASN destabilizes p65 and promotes its degradation in the proteasome.

**Fig. 5:**
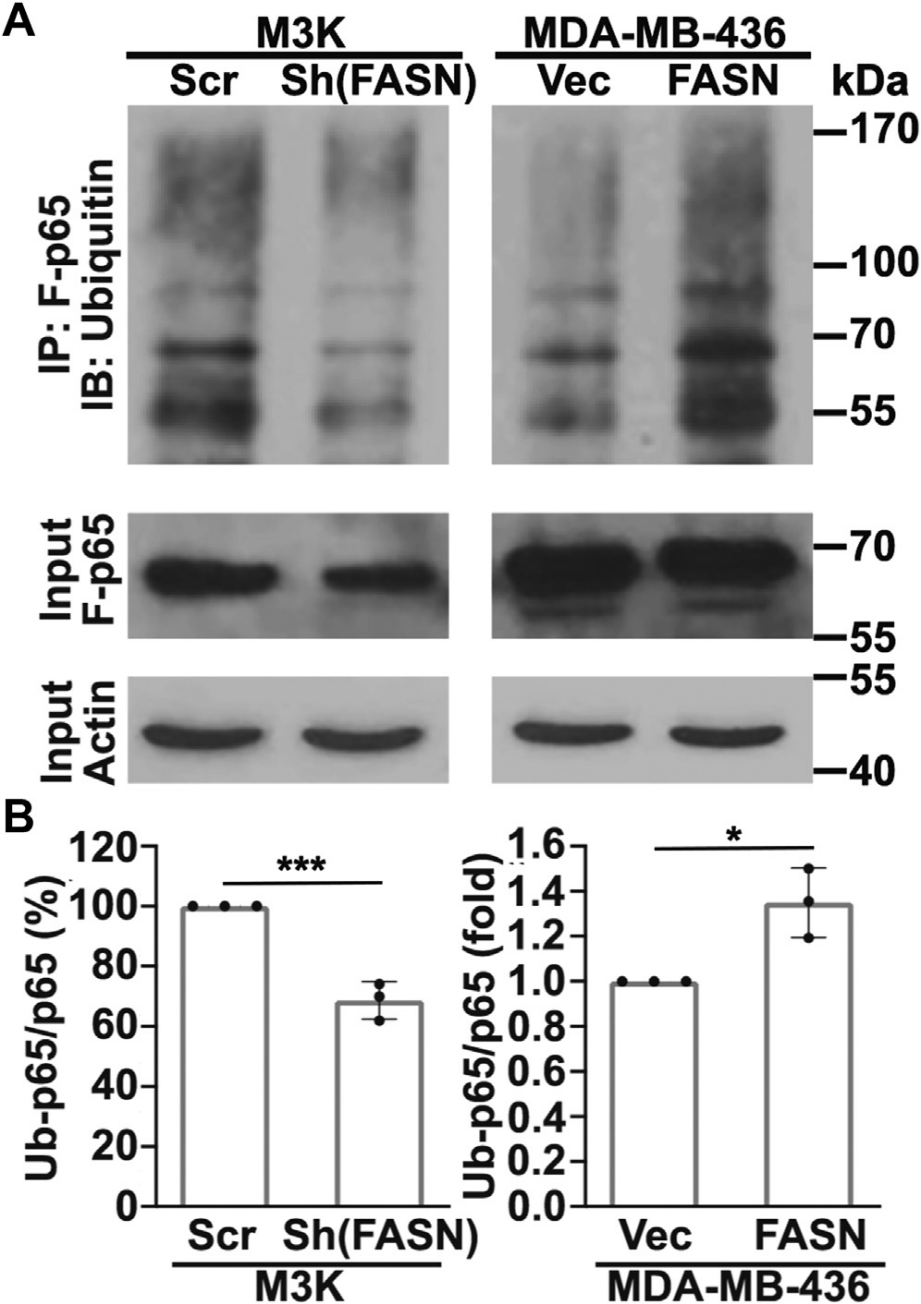
Effect of FASN expression on p65 ubiquitination. A: Western blot analysis. Stable M3K/Sh(FASN) and MDA-MB-436/FASN cells along with their respective control M3K/Scr and MDA-MB-436/Vec cells were transiently transfected with p65-FLAG for 24 h and then treated with 2 μM MG132 for 4 h followed by immunoprecipitation with FLAG antibody and Western blot analysis using α-ubiquitin antibody. Total lysate was used for Western blot analysis of input FLAG-p65. B: Quantification of ubiquitinated FLAG-p65 of from A of three independent experiments (∗*P* < 0.05; ∗∗∗*P* < 0.01).

To provide further evidence that FASN-induced p65 degradation is via the proteasome, we investigated the effect of FASN on ubiquitination levels of p65 given that proteasomal degradation of p65 is linked to its ubiquitination by a variety of different E3 ubiquitin ligases (28). For this purpose, M3K/Sh(FASN) and MDA-MB-436/FASN cells along with their respective control cells were transiently transfected with pcDNA(3.1)-FLAG-p65 to express exogenous FLAG-tagged p65 and treated with 2 μM MG132 to inhibit proteasomal degradation of ubiquitinated p65 proteins. The FLAG-tagged p65 proteins were then immunoprecipitated using FLAG antibody for Western blot analysis of ubiquitinated FLAG-p65. As shown in Fig. 5, FASN knockdown reduced, whereas FASN overexpression increased ubiquitination of FLAG-p65 compared with their respective control cells. Thus, FASN likely destabilizes p65 by stimulating its ubiquitination and degradation in the proteasome.

### Kinase involvement in FASN regulation of p65 expression

**Fig. 6:**
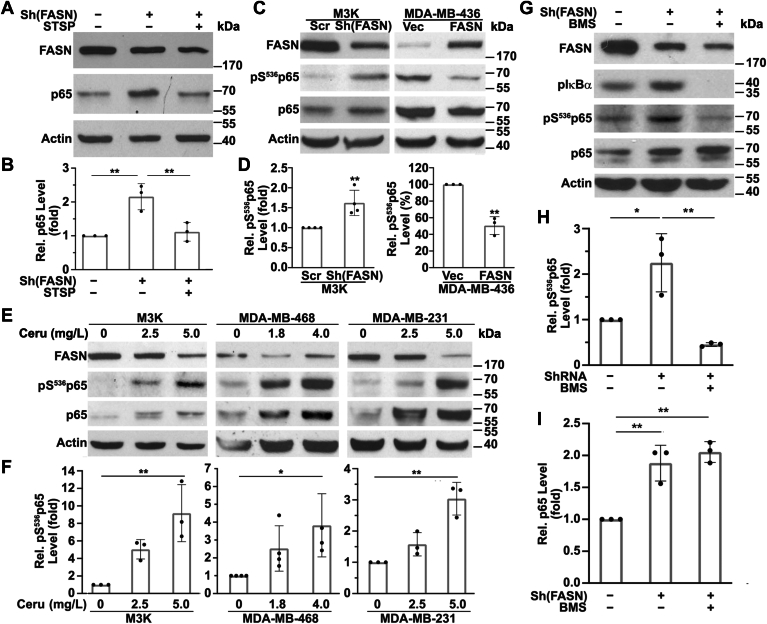
FASN regulation of p65 expression and phosphorylation at Ser^536^. A, B: Western blot analysis of FASN, p65, and actin loading control in stable M3K/Sh(FASN) and control M3K/Scr cells following treatment without or with 100 nM staurosporine for 2 h and stimulation with 50 μg/ml TNF-α for 1 h. C, D: Western blot analysis of FASN, pS^536^p65, total p65, and actin loading control in stable M3K/sh(FASN), MDA-MB-436/FASN, and their respective control M3K/Scr and MDA-MB-436/Vec cells following treatment with 50 μg/ml TNF-α for 1 h. E, F: Western blot analysis of FASN, pS^536^p65, total p65, and actin loading control in M3K, MDA-MB-468, and MDA-MB-231 cells treated for 72 h with increasing concentrations of cerulenin (Ceru) and then with 50 μg/ml TNF-α for 1 h. G–I: Western blot analysis of FASN, pIκBα. pS^536^p65, total p65, and actin loading control in M3K/sh(FASN) cells treated without or with 5 μM IKK inhibitor BMS-345541 for 2 h and then with 50 μg/ml TNF-α for an additional hour. B, D, F, H, I: Quantifications of p65 (B, I) and pS^536^p65 (D, F, and H) from A, C, E, and G, respectively, of ≥3 independent experiments (∗*P* < 0.05; ∗∗*P* < 0.01).

We next attempted to understand the mechanism of FASN-induced p65 destabilization. Previous studies suggested that phosphorylation of p65 at Thr^254^, Ser^276^, and Ser^536^ may be involved in regulating p65 protein stability (29). To determine the potential involvement of p65 phosphorylation in mediating FASN regulation of p65 expression, we first tested the effect of the pan-kinase inhibitor staurosporine on FASN regulation of p65 expression. As shown in Fig. 6A, B, M3K cells with stable FASN knockdown express higher level of p65 compared with control M3K/Scr cells as expected. However, treatment with staurosporine ablated the increase in p65 expression induced by FASN knockdown. Thus, FASN may regulate p65 expression via its phosphorylation.

### FASN regulation of p65 phosphorylation at Ser^536^

We next determined which phosphorylation site in p65 may mediate FASN regulation of p65 stability. Given that Ser^536^ phosphorylation is most commonly associated with NF-κB activation (30) and that this phosphorylation may affect the interaction of p65 with IκBα and potentially its trafficking and stability (31), we first tested if FASN regulates Ser^536^ phosphorylation in p65 by performing Western blot analysis of pSer^536^p65. As shown in Fig. 6C, D, M3K/Sh(FASN) cells express a much higher level of Ser^536^-phosphorylated p65 than control M3K/Scr cells. Consistently, MDA-MB-436/FASN cells exhibit much less pSer^536^p65 than its control MDA-MB-231/Vec cells. Furthermore, treatment of M3K, MDA-MB-468, and MDA-MB-231 cells with cerulenin dose-dependently increased the level of pSer^536^p65 (Fig. 6E, F). Together, these data suggest that FASN likely suppresses phosphorylation of Ser^536^ in p65.

### Ser^536^ phosphorylation does not mediate FASN regulation of p65

To evaluate the possibility that phosphorylation of Ser^536^ mediates FASN regulation of p65 expression, we took advantage of BMS-345541, a kinase inhibitor specific to inhibitor of nuclear factor-kB kinase (IKK), as IKKα/β are the primary kinases known to phosphorylate p65 at Ser^536^ (32, 33, 34). As shown in Fig. 6G, H, 5 μM BMS-345541 treatment of M3K/Sh(FASN) cells successfully inhibited phosphorylation of IĸBα and Ser^536^ of p65, consistent with a previous observation that 5 μM BMS-345541 inhibits IKKα/β activity (35). However, BMS-345541 failed to reverse FASN knockdown-induced p65 expression (Fig. 6I). Thus, Ser^536^ phosphorylation unlikely mediates FASN regulation of p65 stability.

### FASN regulation of p65 stability via phosphorylation at Thr^254^

**Fig. 7:**
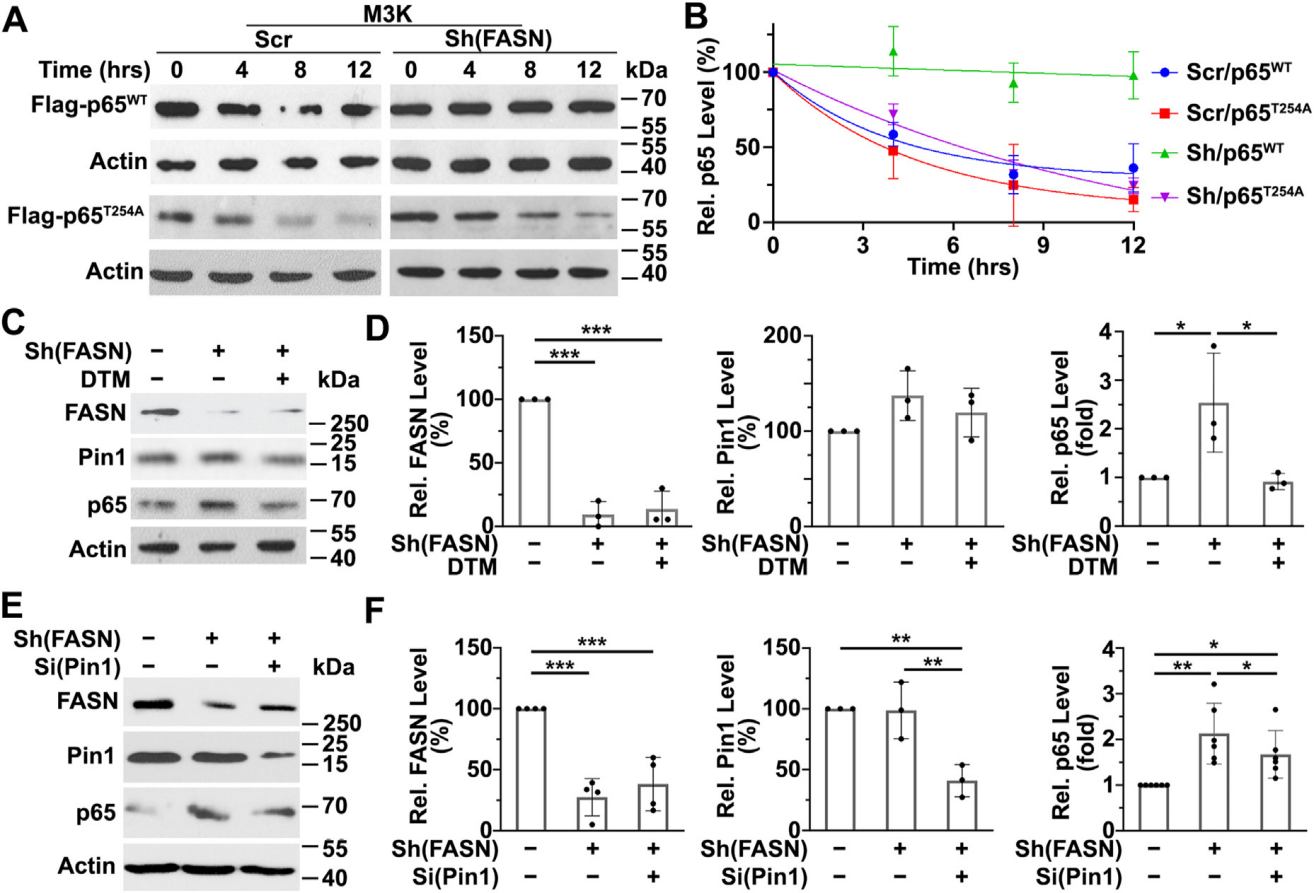
FASN regulation of p65 protein stability and phosphorylation at Thr^254^ and coordination with Pin1. A, B: Half-life of wild-type and Thr^254^Ala mutant FLAG-p65. cDNAs encoding wild-type and Thr^254^Ala mutant FLAG-p65 in pcDNA(3.1) were transiently transfected into M3K/sh(FASN) and M3K/Scr cells and then treated with 60 μg/ml cycloheximide and harvested at the indicated time points before Western blot analysis probed with FLAG antibody. C–F: Effect of Pin1 inhibition (C, D) or knockdown (E, F) on FASN knockdown-induced p65 expression. M3K/sh(FASN) cells were treated without or with 10 μM dipentamethylene thiuram monosulfide (DTM; C, D) or transiently transfected with Pin1 siRNA (E, F) for 48 h followed by Western blot analysis for FASN, Pin1, total p65, and actin loading control. M3K/Scr control cells treated with vehicle (C, D) or transiently transfected with scrambled control siRNA (E, F) were used as a baseline control. B, D, F: Quantifications of p65 from A, C, and E of ≥3 independent experiments each (∗*P* < 0.05; ∗∗*P* < 0.01; ∗∗∗*P* < 0.001).

Another phosphorylation site associated with NF-κB activation and p65 protein stability is Thr^254^ (36). To determine the potential role of Thr^254^ phosphorylation in FASN regulation of p65 expression, we mutated Thr^254^ to Ala in FLAG-tagged p65 and transfected it along with wild-type FLAG-p65 or empty vector control into M3K/Sh(FASN) and control M3K/Scr cells. Subsequently, we determined the stability of these proteins using cycloheximide-chase analysis and Western blot analysis of FLAG-p65. As shown in Fig. 7A, B, the stability of the wild-type FLAG-p65 in M3K/Sh(FASN) cells is considerably increased compared with that in control M3K/Scr cells, which is consistent with the finding that FASN knockdown increases the stability of endogenous p65 (Fig. 4A, B). However, this increase in stability was abolished by mutation of Thr^254^ to Ala. These findings suggest that phosphorylation of p65 at Thr^254^ is likely important for FASN regulation of p65 protein stability.

### FASN regulation of p65 protein stability may involve Pin1

**Fig. 8:**
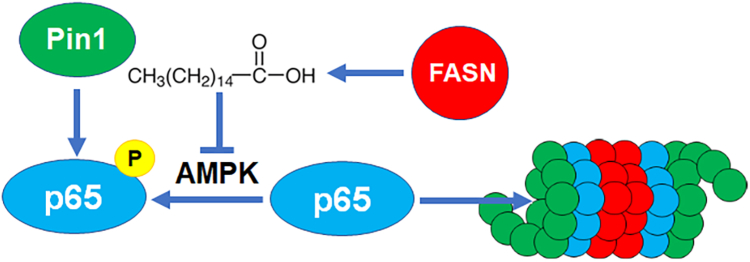
Schematic model of FASN regulation of p65 stability via palmitate and p65 phosphorylation and isomerization by Pin1.

It has been reported that the peptidyl-prolyl *cis/trans* isomerase Pin1 enhances p65 protein stability by binding to the phosphorylated-Thr^254^-Pro motif of p65, and loss of Pin1 disrupts p65 nuclear accumulation and stability (36). To determine the potential role of Pin1 in FASN regulation of p65 stability via Thr^254^ phosphorylation, we used a Pin1 inhibitor, dipentamethylene thiuram monosulfide, and treated M3K/Sh(FASN) cells followed by Western blot analysis of p65. As shown in Fig. 7C, D, dipentamethylene thiuram monosulfide treatment reversed FASN knockdown-induced p65 increase in expression without effect on Pin1 expression or FASN expression. To validate this finding, we transiently knocked down Pin1 and determined the effect on FASN knockdown-induced p65 expression. As shown in Fig. 7E, F, Pin1 knockdown also significantly abated FASN knockdown-induced p65 upregulation. Consistently, Pin1 knockdown had no significant effect on FASN expression. However, Pin1 knockdown also rescued FASN knockdown-induced PARP1 downregulation (supplemental Fig. S5A), consistent with Pin1 function in mediating FASN regulation of p65 and its downstream target gene PARP1 expression. To support the above conclusion, we overexpressed Pin1 in MCF7/FASN cells to determine if it could rescue FASN suppression of p65 expression. As shown in supplemental Fig. S5B, Pin1 overexpression indeed rescued p65 expression from FASN overexpression-induced suppression. Together, we conclude that the isomerase activity of Pin1 likely participates downstream in FASN regulation of p65 protein stability via its Thr^254^-Pro motif, which in turn regulates the expression of p65 downstream target genes, including PARP1 (Fig. 8).

## Discussion

Previously, it was reported that FASN regulates p65 expression, which in turn controls PARP1 expression and cellular response to DNA-damaging treatments (16). Based on these findings, the proton pump inhibitor omeprazole was repurposed to target FASN in a single-arm phase II trial for triple-negative breast cancer (37). Treatment with high-dose omeprazole in combination with the standard-of-care AC-T (doxorubicin and cyclophosphamide followed by paclitaxel) neoadjuvant chemotherapy resulted in much higher pathological complete response rates at 74.4%, compared with AC-T-alone treatments at approximately 40%. In this study, we report that FASN regulates p65 expression not by regulating transcription of the p65 gene but by regulating p65 protein stability via its catalytic product, palmitate, through phosphorylation of Thr^254^ in p65, thereby impacting the ability for Pin1 to bind to and isomerize p65 (Fig. 8).

The findings that inhibiting FASN activity upregulates p65 expression and that addition of exogenous palmitate ablates the effects of FASN knockdown on p65 protein level suggest that likely palmitate as the catalytic product of FASN, not the FASN protein per se, regulates p65 expression. It is noteworthy that FASN knockdown may affect the expression of fatty acid transporters that affect exogenous palmitate uptake indirectly leading to p65 reduction. However, fatty acid transporters such as CD36, FATP3, and FATP4 were undetectable in the breast cancer cell lines used (unpublished observations), indicating that their expression level in breast cancer cells likely had minimal contribution to FASN regulation of p65 expression.

Palmitate is an important precursor for production of structural and signaling lipids and for palmitoylation of cysteine residues known to have a direct effect on the activity of signaling molecules, such as Wnt proteins and Ras GTPases (38, 39). Palmitoylation is also known to affect protein stability (40). However, analyses of several protein palmitoylome datasets (41, 42) failed to show that p65 or any other NF-κB family proteins are palmitoylated. Thus, FASN unlikely regulates p65 stability by producing palmitate culminating in the palmitoylation of p65.

While the finding that FASN regulates p65 stability by impacting the phosphorylation status of the p65 Thr^254^ residue, thereby, affecting the isomerization of p65 by Pin1 (Fig. 8) is intriguing, it is not surprising since Pin1 is known to regulate stability of many proteins by binding to and isomerizing these proteins upon phosphorylation at the consensus Ser/Thr-Pro motif (43) including Thr^254^-phosphorylated p65 (36). It has been reported that activated p65 in the nucleus is not bound by the inhibitory subunit IκBα, leaving Thr^254^ exposed and allowing its phosphorylation (36) followed by recruitment of and isomerization by Pin1. However, it remains unclear what kinase in the nucleus is responsible for the phosphorylation of p65 at Thr^254^. One such candidate kinase that may mediate FASN regulation of p65 phosphorylation is AMP-activated protein kinase (AMPK). It has been shown previously that FASN inhibition activates AMPK (44, 45) and that AMPK activation increases NF-κB nuclear retention and DNA-binding activity (46). It has also been observed that palmitate inhibits AMPK (47, 48, 49). Importantly, AMPK has been shown to localize in the nucleus (50). Thus, it is tempting to speculate that AMPK may be the downstream kinase responsible for FASN regulation of p65 phosphorylation at Thr^254^ (Fig. 8).

Previously, it has also been reported that the classical protein kinase C (PKC) isoform PKCα was activated to phosphorylate IκBα, resulting in its degradation and subsequent activation of NF-κB in response to FASN inhibition by the cerulenin derivative C93 (51). Furthermore, multiple PKC isoforms have been found to be palmitoylated (41, 52). PKC is also known to translocate to the nucleus upon its activation, and some isoforms are resident proteins of the nucleus (53). Thus, PKC may be another potential candidate kinase to mediate FASN regulation of p65 phosphorylation and stability.

Although Pin1 binding to and isomerizing phosphorylated substrate proteins, such as p65 and p53 (36, 43) results in the stabilization of these proteins, the molecular mechanism of the protein stabilization following isomerization is unknown. However, it was thought that phosphorylation and isomerization of these proteins facilitate their translocation into nucleus where they exert their functions as transcription factors, which may prevent them from binding by E3 ubiquitin ligase and degradation by proteasomes in the cytoplasm (36). Whether this is the mechanism of FASN-induced p65 degradation remains to be determined.

It is noteworthy that FASN may also regulate and inhibit phosphorylation of Ser^536^ in addition to Thr^254^ in p65. However, Thr^254^ was identified as the phosphorylation site involved in FASN regulation of p65 stability. Examination of the flanking sequences of these two phosphorylation sites shows that only Thr^254^ is followed by a Pro residue, constituting the consensus binding site for Pin1. However, phosphorylation of p65 at Ser^276^ has been shown to play a role in regulating p65 stability (29). Specifically, Pim-1-mediated phosphorylation of p65 at Ser^276^ prevents ubiquitin-mediated proteasomal degradation of p65 in HeLa cells (54). Whether p65 phosphorylation at Ser^276^ by Pim-1 could also be implicated in FASN regulation of p65 stability remains unknown. Clearly, future studies are necessary to investigate the roles of different phosphorylation sites in FASN regulation of p65 stability.

In summary, we demonstrated here that FASN regulates p65 stability, not its gene transcription, by regulating its phosphorylation at Thr^254^, which recruits the peptidyl-prolyl *cis/trans* isomerase Pin1, an enzyme known to stabilize many proteins in the nucleus. This finding suggests that FASN and palmitate may play an important role in controlling the stability of critical regulators such as p65, which in turn contributes to tumorigenesis and drug resistance. Future studies are necessary to evaluate this possibility as well as potential palmitate regulation of AMPK or PKC activity to mediate FASN regulation of p65 phosphorylation.

## Data availability

All data are contained within the article and Supplemental data.

## Supplemental data

This article contains supplemental data.

## Conflict of interest

The authors declare that they have no conflicts of interest with the contents of this article.
