# Supplementary material for: FASN negatively regulates p65 expression by reducing its stability via Thr254 phosphorylation and isomerization by Pin1
Source: J Lipid Res. 2024 Mar 10;65(4):100529. doi: 10.1016/j.jlr.2024.100529 (PMC11017288; doi:10.1016/j.jlr.2024.100529)
Supplement: Supplemental Figures S1–S5 [file mmc1.pdf]

Supplementary Figures

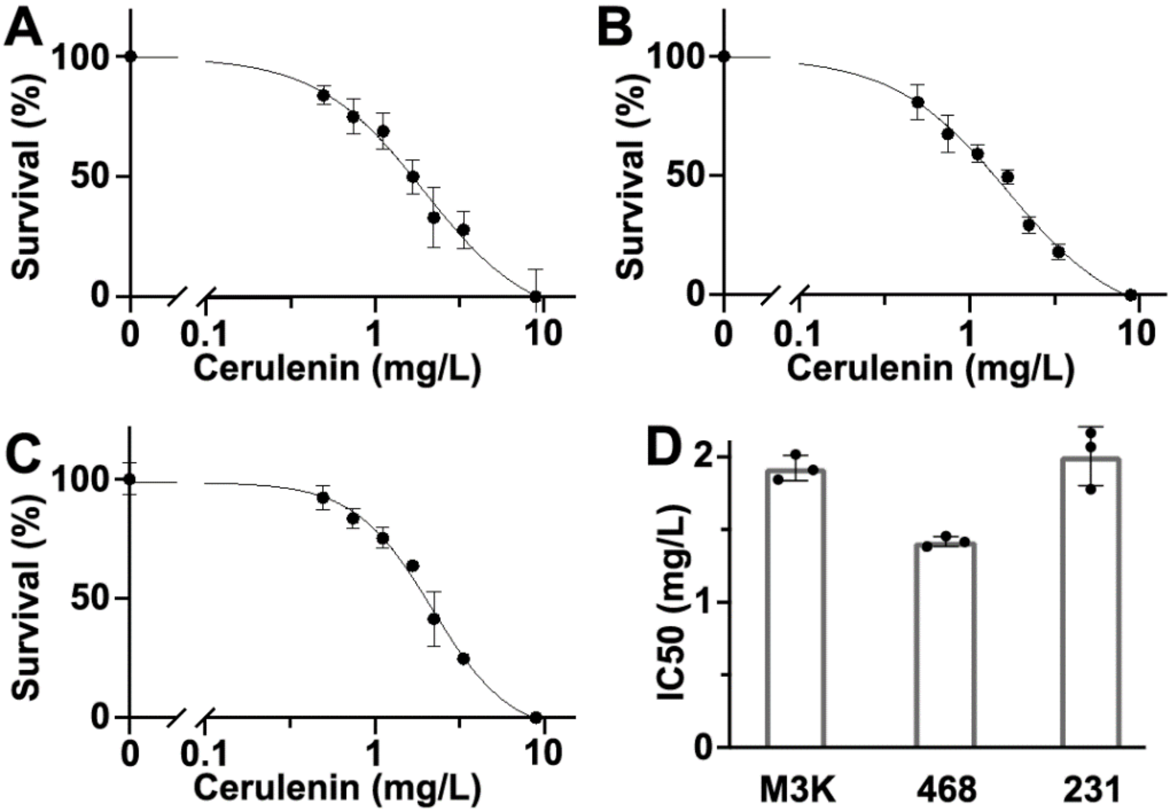

Figure S1. Cerulenin cytotoxicity and IC_{50}. (A-C) Representative cell survival curves determined using methylene blue survival assay in the presence of different concentrations of cerulenin for M3K (A), MDA-MB-468 (B), and MDA-MB-231 (C) cells. (D) Cerulenin IC_{50} in M3K, MDA-MB-468 (468), and MDA-MB-231 (231) derived from survival curves as shown in panels A-C of 3 independent experiments each.

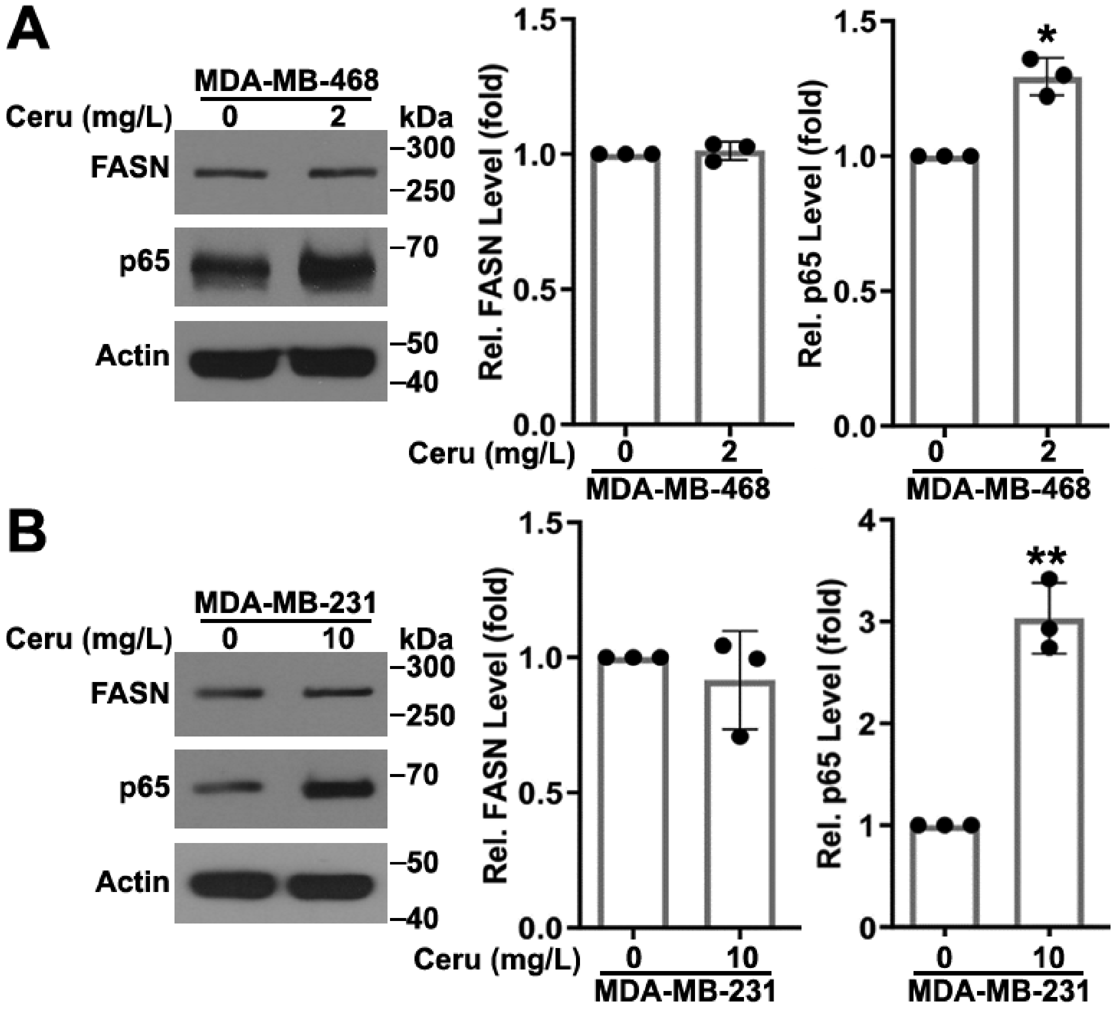

Figure S2. Effect of cerulenin on p65 and FASN expression. Western blot and quantification analysis of FASN, p65 and actin loading control in parental MDA-MB-468 (A) or MDA-MB-231 cells treated without or with cerulenin (Ceru) at concentrations indicated for 2 hours. (*$p<0.05$, **$p<0.01$).

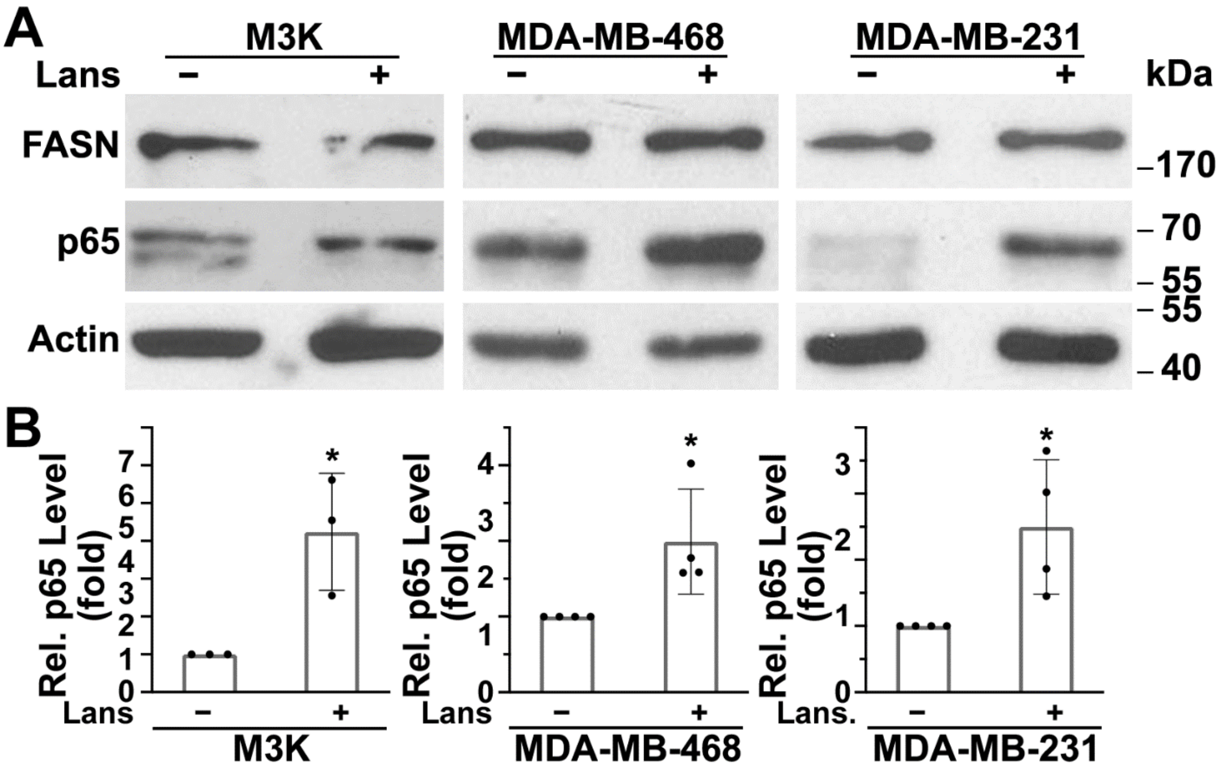

Figure S3. FASN regulation of p65 expression. (A) Western blot analysis of FASN, p65, and actin loading control in M3K, MDA-MB-468, and MDA-MB-231 cells treated with increasing concentrations of cerulenin for 72 hours. (B) Quantification p65 from panel A of ≥3 independent experiments (*p<0.05).

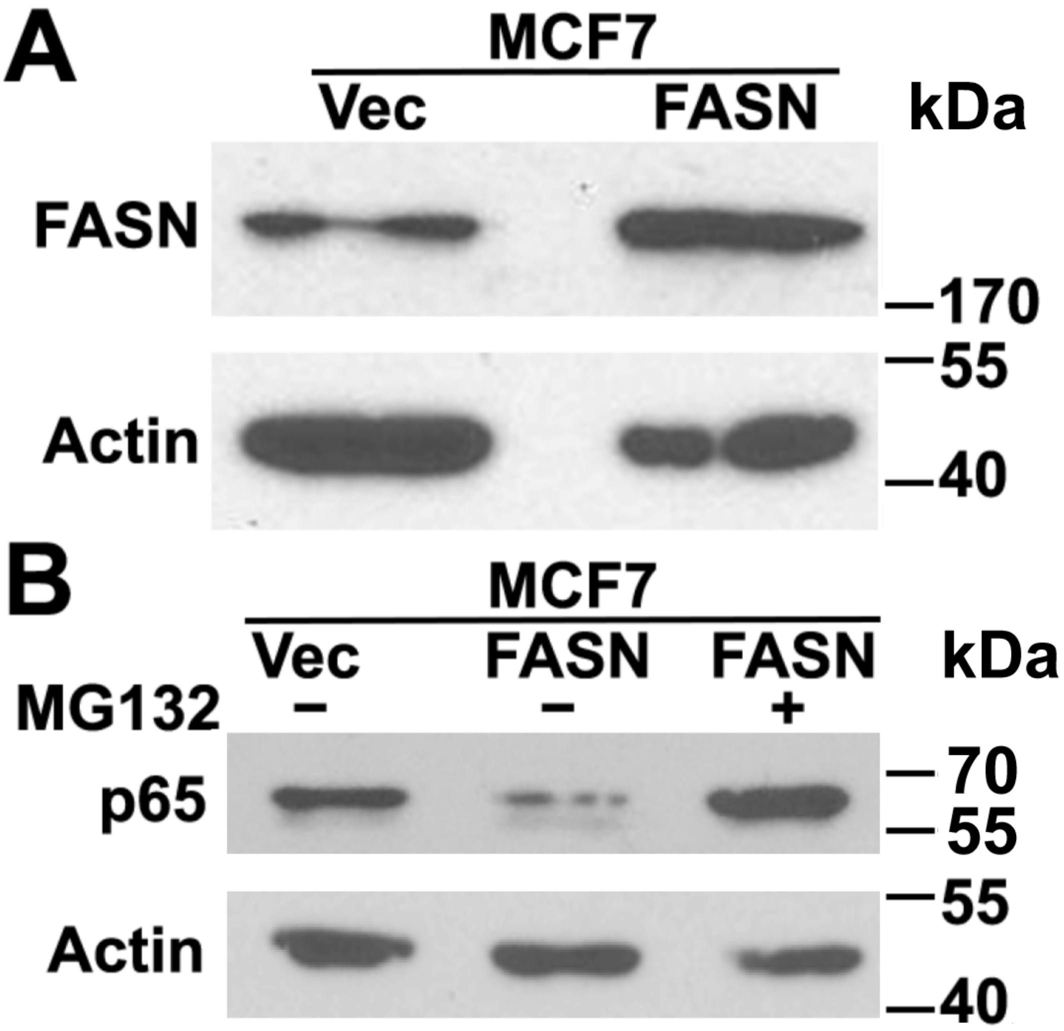

Figure S4. FASN regulation of p65 protein stability. (A) Western blot analysis of FASN and actin loading control in stable MCF7/Vec and MCF7/FASN cells. (B) Effect of MG132 on FASN-induced p65 reduction. Stable MCF7/FASN and the control MCF7/Vec cells were treated without or with 2 μM MG132 for 24 hours followed by Western blot analyses of p65 and actin loading control.

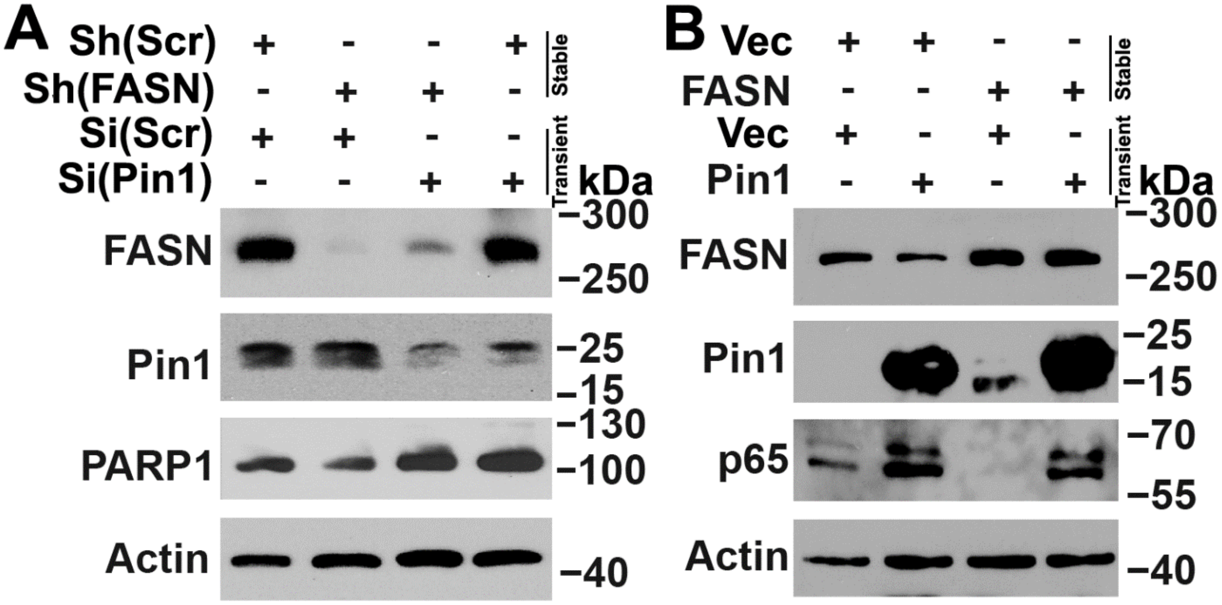

Figure S5. FASN regulates p65 stability via coordinating with Pin1. (A) Western blot analysis of FASN, Pin1, PARP1, and actin loading control in stable M3K/Sh(FASN) and control M3K/Scr cells at 48 hours following transient transfection with Pin1 or scrambled control siRNA. (B) Western blot analysis of FASN, Pin1, p65, and actin loading control in stable MCF7/FASN and control MCF7/Vec cells at 48 hrs following transient transfection with empty pCMV3 vector or pCMV3-FLAG-Pin1 for 48 hours.
